# Stability of Antimicrobial Drug Molecules in Different Gravitational and Radiation Conditions in View of Applications during Outer Space Missions

**DOI:** 10.3390/molecules26082221

**Published:** 2021-04-12

**Authors:** Ágota Simon, Adriana Smarandache, Vicentiu Iancu, Mihail Lucian Pascu

**Affiliations:** 1 National Institute for Laser, Plasma and Radiation Physics (INFLPR), Laser Department, Atomiștilor 409, 077125 Măgurele, Romania; adriana.smarandache@inflpr.ro (A.S.); mihai.pascu@inflpr.ro (M.L.P.); 2 Faculty of Physics, University of Bucharest, Atomiștilor 405, 077125 Măgurele, Romania; vicentiu.iancu@eli-np.ro; 3 “Horia Hulubei” National Institute of Physics and Nuclear Engineering (IFIN-HH), Extreme Light Infrastructure—Nuclear Physics (ELI-NP), Reactorului 30, 077125 Măgurele, Romania

**Keywords:** drug stability, pharmacokinetics/pharmacodynamics, hypergravity, microgravity, spaceflight environment, multiple drug resistance, antibiotics, non-antibiotics, photoactivated drugs, laser irradiation

## Abstract

The evolution of different antimicrobial drugs in terrestrial, microgravity and hypergravity conditions is presented within this review, in connection with their implementation during human space exploration. Drug stability is of utmost importance for applications in outer space. Instabilities may be radiation-induced or micro-/hypergravity produced. The antimicrobial agents used in space may have diminished effects not only due to the microgravity-induced weakened immune response of astronauts, but also due to the gravity and radiation-altered pathogens. In this context, the paper provides schemes and procedures to find reliable ways of fighting multiple drug resistance acquired by microorganisms. It shows that the role of multipurpose medicines modified at the molecular scale by optical methods in long-term space missions should be considered in more detail. Solutions to maintain drug stability, even in extreme environmental conditions, are also discussed, such as those that would be encountered during long-duration space exploratory missions. While the microgravity conditions may not be avoided in space, the suggested approaches deal with the radiation-induced modifications in humans, bacteria and medicines onboard, which may be fought by novel pharmaceutical formulation strategies along with radioprotective packaging and storage.

## 1. Introduction

Six decades have passed since the first human was launched into space, marking the beginning of human space exploration. Humanity’s deeply rooted quest to explore and push the boundaries was boosted further by the Moon-landing. Since the purpose of the early missions was simply surviving, space exploration has evolved to living and working up to performing valuable scientific research in such a harsh environment [1]. Remarkable progress has been made from suborbital to orbital flights, as well as by gradually extending the duration of the missions from minutes to months and their complexity [2]. Currently, the International Space Station (ISS) represents the most advanced permanent man-inhabited orbital project used for cutting-edge scientific and technical developments. Following decades of low-Earth orbit (LEO) space missions, the aspiration to set out for new and even more remote shores has steadily increased, and initiatives have been taken for an international deep space Gateway to the Moon and Mars [3]. However, such lengthy endeavors bring a multitude of challenges, among which microbial infections have serious implications, jeopardizing astronauts’ safety, health and performance, which in turn might compromise a mission. ISS—during its more than 20 years of uninterrupted operation—constitutes an exemplary case for being spatially isolated beyond any other human habitat [4]. Human habitation in confined spaces—whether permanent or temporary—is accompanied by co-habitation with microbes. Especially where air, water, food and waste are recycled, the development of pathogens may exhibit a major risk [5]. One knows that even with the strictest pre-flight quarantine procedures, a multitude of microbe species will contaminate the interior of the spacecraft during extended missions. It has been indicated that spaceflight alters the immune response [5], putting crewmembers in more vulnerable positions in front of opportunistic microorganisms, such as bacteria, fungi and viruses, whose action upon weakened immune functions may lead to severe infectious diseases.

Long-term manned exploratory missions can be differentiated from the ones in LEO in the following approaches [6,7,8]:

- prolonged expedition duration (i.e., Martian expeditions lasting in the order of years);
- extended exposure to space environment;
- lack of restocking capability;
- absence of direct medical or pharmaceutical support from Earth and exclusive use of telemedicine; and
- limited or no capability for emergency evacuation of sick astronauts.

The above-mentioned differences imply rigorous space pharmacology research to be carried out prior to launching such missions, since the use of effective pharmaceuticals with an adequate shelf life (expiration date) will play an essential role in accomplishing extended space voyages. To evaluate whether space environment conditions have compromising effects on medicines, pharmacokinetics (how the drug influences the body), pharmacodynamics (how the body reacts to the effect of the drug) and stability are necessary detailed investigations. This report is centered on drug stability in outer space conditions.

Stability is a quality attribute of drugs and represents their capacity to retain physico-chemical, microbiological and toxicological specifications throughout the duration of storage and use. Stability testing aims to provide evidence on how environmental factors, such as temperature, humidity, gravity and radiation exposure, to name a few, impact the quality of pharmaceuticals over time, as well as to determine their shelf life and appropriate storage conditions [9,10,11].

**Figure 1:**
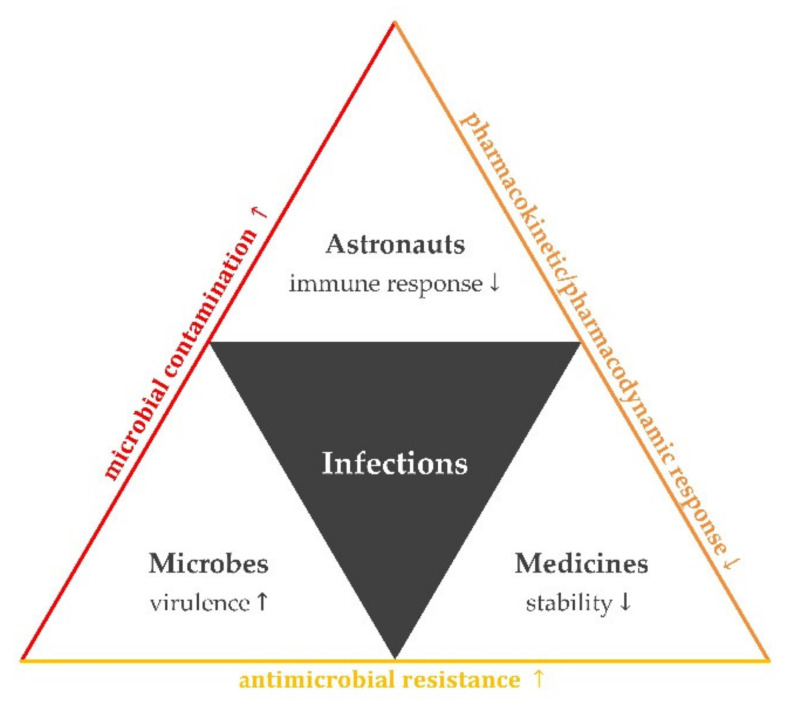
Factors that could enhance the risk of infections and their transmission in the spaceflight environment. Alterations in astronauts’ microbial flora and bacterial physiology could lead to increased microbial contamination onboard. Changes in astronauts’ physiology as well as in physico-chemical properties of pharmaceuticals could influence pharmacokinetics and/or pharmacodynamics. Increased microbial virulence and decreased drug susceptibility could enhance antimicrobial resistance. Notations: ↑ increase; ↓ decrease.

Factors that could impact susceptibility to and severity of infections during space missions are illustrated in Figure 1.

Hence, this review is an overview of the impact of the spaceflight environment on humans, pathogens, as well as existing and new antimicrobial agents, focusing on drug stability in different radiation and gravitational conditions, with the knowledge that stability of medicines contained in space kits is critical for future deep space travel and colonization.

## 2. Impact of Spaceflight Environment on the Human Body

The spaceflight environment is different from what humans are accustomed to on Earth—not to mention that without appropriate protection offered by a spacecraft or spacesuit, it is lethal [12]. It also causes alterations in most physiological processes to some degree, mainly due to the effects of space radiation and the absence of convection, hydrostatic pressure, buoyancy, sedimentation and gravitational loading [2].

Microgravity, for instance, causes fluid shift (from the lower to the upper part of the body) and thoracic expansion that conjointly gives rise to cardiovascular changes, like increased cardiac preload, stroke volume and cardiac output [13], alongside decreased plasma volume [14] and reduced red blood cell (RBC) mass by the destruction of newly produced RBCs [15]. It has been indicated that blood and fluid redistribution can also cause spaceflight-associated neuro-ocular syndrome [16]. The fluid shift and/or the sensory conflict hypothesis can be the underlying cause for space adaptation syndrome [17]. A reduced gravitational environment may deteriorate perception and impair the function of motor skills, reflexes and coordination [18]. Microgravity is liable for musculoskeletal modifications too, such as diminished muscle mass and strength, loss in lean body mass, decreased bone density and significant loss of calcium through excretion, which leads to increased risk of kidney stones [19,20]. Gastrointestinal and metabolic alterations, including changed gastric emptying, increased intestinal transit rate and variations in enzyme activity, have been likewise observed subsequent to spaceflight [20,21]. The list goes on, the above-mentioned effects representing only some of the most relevant ones, which may impact pharmacokinetics, pharmacodynamics or both during space voyages, as reviewed in more detail in References [20,21,22,23]. In such cases, the sought pharmacotherapeutic results may not be attained in space with dosing regimens established in terrestrial gravity.

Apart from the inherent effects of external and internal physical stressors of space missions, psychological and social-interpersonal stressors have been identified as well [24]. The isolated and confined environment of spacecraft may produce sleep disturbance, circadian desynchronization, deprivation (from people outside the habitat) and separation response (from Earth) of crewmembers—to name a few of the most significant ones. This is likely to lead to mental health and behavioral disorders and may turn out to be of paramount importance in long-term deep-space ventures [24,25,26,27]. Therefore, dedicated research is continuously performed in space analogs, including Antarctic research stations, polar expeditions, undersea habitats and space simulator experiments [25,27,28,29].

It has been indicated by Guena et al. that high levels of chronic stress may impact the immune system [24]. Immune dysfunctions in returning astronauts have already been noticed, dating back to Apollo and Skylab missions [30,31]. Later in vitro studies have revealed that disturbances of the immune system are gravity-dependent, the microgravity environment of spaceflight causing a drastic decrease in lymphocyte proliferation rates [32]. More recently, latent herpes virus reactivation has been evidenced during space missions, Epstein-Barr virus [33,34], varicella-zoster virus [35] and cytomegalovirus [36] being able to reactivate individually or in combination [37], and serving consequently as a tool for the detection of downregulation of astronauts’ immunity [36].

Guéguinou et al. outlined several results obtained on the effect of spaceflight on organs of the lymphoid system and its cell populations, spleen, thymus and lymph node hypoplasia being observed in many investigations [5]. Since the manipulation of germs—constricting the exposure, colonization, invasion and infection—relies on the well-functioning of cells involved in the immune system, spaceflight-associated hypoplasia in lymphoid organs induces considerable consequences [8]. The outcomes regarding leukocyte populations in human and animal studies subjected to spaceflight are contradictory, one of the explanations being that immunological modifications may be connected to mission duration [38]. Parameters, such as number, function and development of cellular components of innate and adaptive immunity are restrained under spaceflight conditions, as documented via studies reviewed in Reference [5]. Quantitative and functional changes in granulocytes (e.g., neutrophils [39]), monocytes [40,41] and natural killer (NK) cells [42,43,44], for instance, have been noticed when comparing before launch and after landing peripheral leukocyte distribution, phagocytic, oxidative burst and cytotoxic activity. Considering that interferon generation triggers the “first line of defense” to help eradicate viral infections and its alteration presents further evidence of the impaired innate immunity during spaceflight [5], the so-called Interferon experiment was conducted aboard Salyut 6. The results revealed a drop in induced interferon production and NK cell activity in astronauts’ lymphocytes when returning from short-duration missions [45]. The well-established Drosophila model of the human innate immunity with its Toll and Imd signaling pathways—regulators of the antibacterial and antifungal defense response—was also used to observe whether different gravitational conditions induce modifications in the immune response [46]. Taylor et al. disclosed that in hypergravity survival of flies infected with *Beauveria bassiana* has increased. Within their subsequent experiment, flies were grown in microgravity during Space Shuttle STS-121 mission and infected with the same pathogenic fungus or *Escherichia coli* upon their return to Earth. Results showed that the Toll pathway is dysfunctional, while the Imd is activated normally [46]. Changes under low-g conditions in the adaptive defense—composed of the cell-mediated and humoral response—have been reported as well [5]. There is evidence of the activation of T lymphocyte cells being depressed in microgravity [47]. The effect of spaceflight conditions on immunoglobulin gene expression was also studied within the Genesis experiment performed on Mir during the Perseus mission [48]. A differential sensitivity between cellular and humoral response under the spaceflight environment has been noted, the cellular response being affected by short-term, while the humoral response by long-duration missions [5]. Nevertheless, one must not neglect the effects induced by space radiation on T cells. The investigations carried out on Space Shuttle STS-118 mission revealed that 30 out of 84 cancer-related genes found in the thymuses of mice suffered considerable modifications as a result of spaceflight exposure [49]. Moreover, the humoral response may be modified by radiation experienced throughout space missions, reduced number of IgG antibodies in mice subjected to a low dose of gamma radiation being evidenced, for example [50].

The risk of infectious diseases may be significantly increased, considering immune dysregulations encountered during space missions. For instance, approximately half of the crewmembers of the Apollo era have reported minor episodes of microbial infections [51]. Since the astronauts’ bacterial flora represents the predominant source of onboard contamination, in-flight cross-infection with potentially pathogenic microbes between crewmen was also documented early [52], alongside changes in the gastrointestinal bacterial community on Apollo missions [51], Salyut and Mir stations [53]. Bacterial species, like *Staphylococcus* and *Corynebacterium* species, associated with human skin and mucous membranes, dominated the ISS environment [54].

Hazards of space travel can be—to a certain extent—compensated with countermeasures that may demand strenuous and time-consuming exercises, as well as pharmacological treatment [8]. Even so, some affection might persist after the return to Earth.

## 3. Terrestrial Microbial Concerns: Approaches for Multiple Drug Resistance

Multiple drug resistance (MDR) of infectious agents is currently responsible for over 700,000 deaths yearly on Earth, with an exponential rise trend to above 10 million deaths annually by 2050 and economic costs estimated at $100 trillion [55].

Researchers continue to investigate different approaches to slow down the rise of MDR in the absence of new antibiotics, whose development requires consistent investments, in terms of time, finances and professionals. One of the strategies consists in drug repurposing, which involves identifying new pharmacological uses of previously approved drugs, other than those corresponding to the initial medical indications [56]. Thus, the combination of antibiotics with non-antibiotics as enhancers contributes to extend the lifetime of antimicrobial agents and combat MDR. A representative case is the efficacious and extensive administration of β-lactam antibiotics, such as amoxicillin in association with β-lactamase inhibitors, like clavulanic acid. These compounds are incorporated into the well-known Augmentin^®^ broad-spectrum antibiotic drug, which successfully treats complicated and resistant bacterial infections [57,58,59,60]. In 2016, the US Food and Drug Administration (FDA) approved avibactam, the first member of a new family of β-lactamase inhibitors. This is a diazabicyclooctane (DBC) with strong inhibitory activity on some enzyme classes, hence ensuring protection against resistance mechanisms associated with β-lactamases [61]. A combination of avibactam with ceftazidime, a third-generation cephalosporin, is marketed as Avycaz^®^ [62]. Other DBCs are currently in various stages of development, vaborbactam being recently approved by FDA as a novel β-lactamase inhibitor [63]. Vaborbactam, in combination with meropenem (an antibacterial agent of the carbapenem class), is available under the name Vaborem^®^, previously called Vabomere^®^ [64,65]. In an effort to identify antibiotic adjuvants, Chevalier et al. assessed 17 quinazoline derivatives to determine whether they can reverse MDR. They found that several investigated samples enhance the antibiotic susceptibility of a panel of MDR Gram-negative isolates, N-alkyl compounds leading to greater activity of chloramphenicol, sparfloxacin and nalidixic acid [66].

To implement antimicrobial adjuvants during space missions, one has to first undertake comprehensive terrestrial investigations, primarily in terms of their stability.

A stability assay of a quinazoline derivative was determined based on UV-Vis absorption spectra measurements. The compound, generically named BG1188, shows preserved absorption characteristics when dissolved in ultrapure deionized water in the concentration range 10^−3^ M–10^−5^ M. Apparently, the storage temperature has no considerable influence on the stability of investigated solutions for up to one month. Samples having a 10^−3^ M concentration are stable within the first month after preparation when stored in a dark environment at 4 °C, while those diluted down to 10^−5^ M maintain their stability even up to three months [67]. Amine derivatives of imidazolidine-4-ones have been evaluated as well, including their capability to boost the effectiveness of antibiotics against *Staphylococcus aureus* ATCC 25923 reference strain and resistant clinical isolate methicillin-resistant *S. aureus* (MRSA) HEMSA 5. The naphthalene compound, in combination with β-lactam antibiotics and ciprofloxacin, proved to be the most potent against the resistant strain, while the anthracene compound also exhibited efficacy enhancement in oxacillin [68]. A stability study in the function of storage temperature and illumination conditions has been carried out by Smarandache et al. [69] for novel thio-hydantoin derivatives, generically called SZ-2 and SZ-7, which exhibit promising pharmaceutical properties [70]. The investigation highlights that SZ-2 samples present instability within the first two days, preserving their absorption characteristics afterwards if kept in the dark at 4 °C. As far as storage in ambient light conditions at 22 °C are concerned, they undergo continuous and accelerated degradation. The stability of SZ-7, stored in the dark at 4 °C, remained unchanged for up to four days, while in the ambient light environment at 22 °C, it was unmodified for only one day [69].

Studies, performed on various non-antibiotics, brought to light their bactericidal effects. For instance, ebselen (EB) and 5-fluoro-2′-deoxyuridine (FdUrd) have been associated with potent antibacterial properties in fighting Gram-positive pathogens, in particular *S. aureus* clinical isolates with enhanced MDR [56]. EB is an organoselenium compound considered as a multifunctional agent, exhibiting in vitro and in vivo antimicrobial activity [71,72,73,74]. FdUrd, an antineoplastic agent, inhibits the thymidylate synthase and along with its metabolites affects RNA synthesis by replacing uracil [75]. EB and FdUr presented minimum inhibitory concentration (MIC) values under micromolar concentrations when used on clinical isolates of MRSA, vancomycin-intermediate and vancomycin-resistant *S. aureus*, showing an elevated survival rate for mice when they were infected with lethal septicemic MRSA [56]. The possible clinical applications of non-antibiotics combined with tetracyclines have been considered as well, one case being the synergistic activity of minocycline antibiotic with anti-diarrheal loperamide drug [76].

Studies in the past decades on phenothiazines, primarily used as antipsychotic drugs, but also known for their multifunctionality, have disclosed that not only do they enhance—in particular conditions—the effect of antibiotics, but may also have inherent antibacterial properties and therefore may be of help in combating MDR [77]. It has been indicated that phenothiazine derivatives, including fluphenazine, thioridazine (TZ), perphenazine and chlorpromazine (CPZ), can interfere with DNA replication [78,79], may influence cellular energy generation [80,81] and inhibit biofilm production [82,83]. Phenothiazines, as efflux pump inhibitors, are of interest because they may be able to augment the antimicrobial activity of available antibacterial agents and increase their intracellular concentration [84,85,86]. Unfortunately, the clinical use of these compounds is limited due to their cytotoxicity. Thus, understanding the mechanisms of phenothiazines activity against bacteria could allow for the development of more active and less cytotoxic efflux inhibitors [87].

Stability studies were carried out based on the UV-Vis absorption spectra of 10^−5^ M–10^−3^ M aqueous TZ solutions, preserved at 4 °C in the dark, 22 °C in dark and 22 °C in ambient light environments. The generation of sulfoxide derivatives is one possible explanation for spectral modifications experienced by TZ samples exposed to ambient light [88]. Investigation into promethazine (PMZ) stability was undertaken in the same manner in Reference [89] as well. Samples kept in a dark environment showed slow degradation as a function of time due to the decline of the molar attenuation coefficient associated with the specific absorption peaks, whereas those stored in ambient light conditions presented significantly faster degradation, accompanied by changes in UV-Vis absorption spectra due to PMZ metabolites, such as PMZ sulfoxide, desmonomethyl PMZ and desmonomethyl PMZ sulfoxide [89].

In the same line of drug repurposing [56,90,91,92] stands the transformation of molecular structures of medicines by exposure to laser radiation as part of a new strategy in chemistry, biology and pharmacology. By selecting the proper irradiation parameters, such as laser wavelength, exposure time and dose, one can control the generation of photoproducts and their concentrations [93]. Therefore, the use of TZ as an antimicrobial drug by exposing it to pulsed laser radiation emitted at 266 nm was reported in Reference [94]. Increased antimicrobial activity has been observed for irradiated TZ when tested against Gram-positive strains, the best effect was obtained for 120 min laser exposed TZ with MIC values 4–16 times lower, compared to unirradiated TZ. Among the investigated strains, *S. aureus*, including the ciprofloxacin-resistant strains, was the most susceptible infectious agent to the enhanced antimicrobial activity of TZ probably due to target protein mutations (in the case of *S. aureus* SM1) or increased efflux (for *S. aureus* ATCC 25923 EtBr). Against *S. epidermidis* ATCC12228_EtBr, 60 min exposed TZ turned out to have the same improved antimicrobial property. A time stability study of unirradiated and irradiated TZ performed via UV-Vis spectroscopy found that all laser exposed samples stabilize after 24 h, except 1 min and 5 min irradiated solutions that were stable only after 48 h. Both 180 min and 240 min exposed TZ were then stable for 1 week. TZ samples irradiated between 1 min and 120 min were stable for two weeks, while unirradiated TZ was stable for up to three weeks. During the first 24 h–48 h following irradiation, new transient compounds are evidenced in UV-Vis absorption spectra like those responsible for the peaks at 454 nm and 486 nm generated in the first 15 min of laser exposure alongside the peaks at 637 nm and 882 nm in samples exposed to laser radiation ranging from 1 min to 120 min [95]. In Reference [96], a cytotoxicity investigation of newly generated molecules via laser exposure—as possible novel therapeutic agents—was described along with an in-depth analysis of the stability of phenothiazine solutions to identify the time frame within which laser-irradiated samples are stable and may be implemented in biomedical applications. The results highlighted dissimilarities in cell viabilities depending on the utilized endpoint assay and cell line type; however, the most significant differences in phenothiazine derivatives, such as CPZ, TZ, PMZ and promazine (PZ), were noticed to be related to the irradiation process. A similar observation regarding the importance of irradiation parameters stands out in the work of Andrei et al., where results on CPZ microdroplets and bulk solutions were examined after interacting with 266 nm and 355 nm pulsed laser radiation. Both, microdroplet and bulk samples, subjected to the laser beam emitted at 355 nm underwent faster molecular modifications when compared to the 266 nm exposure case [97]. Returning to the previous topic, photoproducts obtained upon laser interaction with CPZ—of which some major compounds have been identified as PZ, hydroxy-PZ, PZ sulfoxide, 2-hydroxy-PZ sulfoxide and CPZ sulfoxide [98]—and PZ displayed increased in vitro cytotoxicity against cell cultures. This suggests that such a strategy may prove beneficial for the development of enhanced bioactive compounds with respect to the original ones. The stability of irradiated phenothiazine solutions in time was established based on their spectral behavior, measured by UV-Vis absorption and FTIR spectroscopy, together with microfluidic properties via surface tension measurements. For instance, the photoproducts mixture of irradiated CPZ and PZ solutions—considered to be the most efficient derivatives used on cell cultures—were not stable within the first 24 h following laser exposure; however, after that time interval, they became stable. The existence of transient compounds in solutions did not affect the results of the study, since it was carried out after the instability period [96]. Another report has shown that 15 min and 30 min laser exposed CPZ samples are highly efficient against methicillin-susceptible *S. aureus* (MSSA) ATCC 6538, MSSA ATCC 25,923, MRSA ATCC 43,300, *Enterococcus faecium* 17-VAR, *E. faecalis* ATCC 29,212 and *Bacillus subtilis* 6633 Gram-positive strains, in both the planktonic and biofilm-growing state as well [99]. One of the most recent studies [100] described enhanced antimicrobial activity by using irradiated CPZ against *S. aureus*, *E. faecalis*, *E. coli*, *Pseudomonas aeruginosa* and *Candida parapsilosis*. Molecular docking evidenced that the action of photoproducts mixture leads to the increased inhibitory effect of the laser exposed CPZ. Biofilm formation on CPZ-impregnated catheters by *P. aeruginosa* and MRSA has also been reduced [100].

The specific characteristics of light that allow for the manipulation of antimicrobial properties of photoresponsive drugs represent valuable means to tackle MDR-associated issues. Recent research introduced photoswitchable antibacterial agent analogs, revealing their selective on/off “light switching” behavior [101,102,103,104].

The emergence and spreading of MDR could not only be of terrestrial concern, but also in space missions, especially long-duration ones. The spaceflight environment alters microbial responses, including enhanced resistance to conventional antimicrobial treatments, as presented in Section 4. Therefore, spaceflight studies might offer valuable insight into the fundamental mechanisms leading to resistance acquisition, which could be of interest on Earth as well. Terrestrial research, in turn, might provide a better understanding of space-associated microbial responses.

## 4. Spaceflight Microbial Concerns: From Bacterial Virulence and Antibiotic Susceptibility to Evidence for Microbial Contaminations

Taking into consideration that bacterial pathogens have evolved so that they may survive even in the most hostile environmental conditions due to their innovative adaptive strategies [105]—as a component of natural selection—numerous space microbiology experiments have been performed, starting with Korabl-Sputnik 2 and Discoverer 17 satellites as early as 1960 [106]. *E. coli* and *Salmonella typhimurium* launched in 1967 aboard Biosatelitte 2 and cultured in orbit presented increased growth [107]. During Skylab 2, 3 and 4 missions, the overall number of aerobes bacteria has raised [108]. The Cytos experiment also involved studies of cell proliferation kinetics under the influence of the spaceflight environment [109,110].

Since those earlier investigations, it was found that bacteria not only survive, but also thrive in certain conditions encountered during spaceflights. For instance, microgravity, hypergravity, vibration and radiation may be associated with several changes in bacteria compared to their terrestrial controls. The microgravity environment is considered to act as the dominant influencing factor when it comes to bacterial growth kinetics and cell behavior, but space radiation may be responsible for the increased mutation rates in microbes [8]. A multitude of in vitro studies showed that spaceflights have a “stimulating effect” on bacterial cultures. Rigorous reports present discrepancies between terrestrial and in-flight responses, like lag phase shortening, growth rate speed-up, final cell population density increase, exponential phase extension, antibiotic sensitivity reduction and transfer increase of genetic material through conjugation, as enlisted by Benoit et al. [111]. All these changes lead to the assumption of secondary metabolite productivity increase. Even though the early theoretical studies have predicted that gravitational forces do not induce any changes in bacteria due to their small size [112], it was suggested later on that the microgravity environment of spaceflight can indirectly affect bacteria besides influencing factors such as cell motility and growth medium [113]. A detailed explanation regarding the correlation between microgravity-induced growth differences and cell motility/culture medium type is given in Reference [8]. The Shenzou 8 spacecraft flew 15 species of microorganisms. Su et al. indicated that all the bacterial species experienced alterations in amino acid and glucose metabolism along with antibiotic-associated secondary metabolites production [114]. Stimulation of microbial antibiotic production has been investigated, first, onboard Space Shuttle missions and then on ISS. Within STS-77, *Humicola fuscoatra* parasitic fungus was used to produce the antifungal agent monorden, greater specific productivity being obtained compared to ground control samples [115]. The generation of antibiotic and anticancer compound actinomycin D by *Streptomyces plicatus* onboard STS-80 and STS-95 resulted in a higher specific yield than that achieved in terrestrial specimens. The agent itself was not affected, although its production time course in space varied depending on the growth medium [111,116]. To obtain information over the full-time course of productivity, another experiment was conducted during ISS 8A with the same bacterium and the results only exhibited greater actinomycin D productivity in the early stages of spaceflight [111]. The explanation may reside in the reduced lag phase in which case cultures step into the growth phase sooner, and in doing so, they may attain the production stage faster [111].

Zea et al. performed an extensive literature review regarding bacterial growth and antibiotic effectiveness research run in space during the last half-century, *E. coli*, *B. Subtilis*, *P. aeruginosa* and *S.* *typhimurium* being amongst the most frequently studied species [117]. Decreased susceptibility of bacterial pathogens to antimicrobial agents has been continuously observed during space missions. The Cytos 2 experiment, carried out on board Salyut 7, examined the sensitivity of *S. aureus* to oxacillin, chloramphenicol and erythromycin alongside *E. coli* to colistin and kanamycin [118]. It was followed by the Antibio experiment during Spacelab D1 mission within the Biorack program [119] and the one during the Space Shuttle STS-42 mission within the IML-1 program [120], both studying *E. coli*, the former (D1) in interaction with colistin, while the latter (IML-1) with dihydrostreptomycin. The results evidenced the accelerated growth rate of *E. coli* with antibiotics [120] and its enhanced antibiotic resistance by adapting itself to grow at higher antibiotic concentrations in space compared to ground samples [118,119,121,122]. The increased MIC associated with different antibacterial compounds in microgravity [118,121,123] is to some extent caused by cell wall thickening [120,124]. One of the first transcriptional and genomic analyses clarified the governing molecular modifications induced in bacterial cells by environmental signals originated from microgravity growth conditions. *S.* *typhimurium* grown during the Space Shuttle STS-115 mission enhanced virulence in a murine infection model in contrast to identical ground control cultures and displayed expression changes in 167 transcripts and 73 proteins [125]. Afterwards, virulence-related genes induced during spaceflight-growth were found in *P. aeruginosa*, and the RNA-binding protein Hfq was brought up as a global regulator of the microbial response to spaceflight [126]. A recent study—complementing previous investigations [127,128] performed within the same Antibiotic Effectiveness in Space experiment on ISS about *E. coli*—showed changes in the expression of 63 genes, including oxidative stress and starvation response, concerning raised gentamicin concentrations in terrestrial as well as spaceflight conditions [122]. Aunins et al. identified 50 upregulated stress-response genes as a reaction to the microgravity environment relative to the equivalent gentamicin concentrations in matched ground controls, revealing a cross-over of stress responses, which justifies the increased antibiotic tolerance in space [122].

Nevertheless, it is imperative not to disregard the effects of hypergravity. Considering that all organisms on Earth have developed and evolved under terrestrial gravitational conditions, subjecting them to high levels of gravity may cause significant alterations in their structure and/or behavior. In high-g conditions, pathogens are likely to survive, grow and even proliferate [129]. The increased gravitational environment can be encountered during launches, re-entries and landing of spacecraft as well as at ejection of rock fragments into space from a planet of origin and their atmospheric entry to the destination planet, for example. In line with the model prediction of Reference [130], during a hypothetical rock ejection from Mars, a hypergravity level of 3.8 × 10^5^ g would be achievable. Therefore, centrifugation and ballistic tests have been performed in extreme acceleration up to 4.5 × 10^5^ g, showing a high survival rate of *Bacillus* spore-forming bacteria species [130,131]. Earlier research has revealed that, after being centrifuged at 4.5 × 10^5^ g for 24 h, *E. coli* exhibited a survival rate of 38.5%, as determined by their ability for colony formation. The survival rate of *Saccharomyces cerevisiae* was almost 100%, ascertained by the methylene blue reduction test [132]. However, investigation at such hypergravity levels of microbial proliferation, and not only simple survival, sheds light on the limits of the organisms’ viability, *Paracoccus denitrificans* and *E. coli*, for instance, being able to proliferate even at ~4.04 × 10^5^ g, as demonstrated in Reference [129].

Along with observations of enhanced virulence in microgravity, cellular aggregation and extracellular matrix accumulation have also been noticed in spaceflight-grown bacteria, being consistent with biofilm formation [125]. Biofilm-associated contaminations onboard space stations were identified as early as Salyut(s), Skylab and Mir stations, bacterial and fungal biofilms being inclined to exhibit enhanced resistance against disinfectants, antibiotics and environmental stresses [133]. Biofilm formation was assessed during Space Shuttle STS-81 mission, showing five times higher biofilm plate count for spaceflight water-grown *Burkholderia cepacia* compared to terrestrial controls, concluding that such an environment enhances bacterial growth and diminishes disinfectant sensitivity [134]. Another early study on biofilm formation in space was conducted on *P. aeruginosa* within the Space Shuttle STS-95 mission [135]. More recent works carried out within STS-132 and STS-135 missions evidenced that *P. aeruginosa* biofilms, which were formed during spaceflight, exhibited an increase in the number of viable cells, biomass and thickness as well as a novel architecture relative to identical Earth controls [136]. The latest investigation by Zea et al. aimed to characterize biofilm production, grow and gene expression in microgravity on different materials [133], more data on this topic being reviewed in References. [137,138]. The relevance of such research has been signaled when microbial contaminations were reported onboard space stations. It has been revealed in Reference [139] that during its 15 years of operation, the orbital station Mir has developed a microbiome of 234 species (108 of bacterial and 126 of fungal flora), bacterial isolates being also identified in drinking and recycled water [140]. Although, not only in the case of former missions and space stations but onboard ISS as well, environmental biocontamination has been found. Novikova et al. carried out a comprehensive survey over six years, concluding that *Sphingomonas* and *Methylobacterium* species contaminated predominantly potable water, *Staphylococcus* was the prevalent bacterial genus in air and surface samples, *Aspergillus* and *Penicillium* species were the preponderate fungi in air samples, while *Aspergillus* and *Cladosporium* species dominated on surfaces [54]. Species of the human microbiota, besides the continuously changing crew of ISS, should not be disregarded as they can be considered to be the most likely source of contamination aboard [54], as discussed in Section 2.

Finally, one must not neglect the effects of space radiation on microbes, since based on the radiation source and its energy—upon interacting with DNA—it may induce reversible or irreversible damages. In turn, this can potentially alter the genetic code via mutations and even passing them to next generations, examples of space radiation effects on responses of microbial mechanisms being provided in Reference [141]. The mutation frequencies increased two to three folds in cloned genes carried by *Saccharomyces cerevisiae* yeast flown 40 days on Mir station compared to terrestrial controls, gene deletion in spaceflight mutant samples being also observed most likely due to space radiation [142].

Reference [143] may be consulted for supplementary information concerning microgravity and radiation-induced effects on microbes.

## 5. Impact of Spaceflight on Drug Stability: From Radiation to Gravitational Environment

The outer space radiation environment represents a combination of charged particles originating from galactic cosmic radiation (GCR) having energies between 100 MeV/n and 10 GeV/n, solar particle events (SPE) with energy reaching several GeV and particles—primarily electrons and protons—trapped by the magnetic field of Earth with an average kinetic energy of a few hundred keV in the case of electrons and hundreds of MeV for protons [144,145,146,147,148]. The high Z energetic particles—constituents of GCR—can induce significant damage when absorbed by spacecraft structures, depositing more energy per unit path depth and generating upon interaction with materials (through fragmentation process) secondary sources of radiation [145,147]. Moreover, a possible contributor to tissue damage and carcinogenesis may be the high ionization power of GCR radiation [144,146]. In the case of occurrence, exposure to SPE would supplement the nominal radiation dose inside the spacecraft pertaining to GCR nuclei and is expected to reach ~0.028 mGy/h during interplanetary space missions. Actually, this value would be modified by a set of parameters, like peak flux, energy spectrum and duration of SPE, alongside characteristics of the shielding material of vehicles [147]. Even though inner-trapped electrons can be impeded by a minimum shielding to reach the intravehicular space, they still pose significant risks regarding astronauts’ extravehicular activities or externally located slightly protected devices. However, trapped protons can penetrate shielded vehicles or stations, playing a role in the radiation exposure of crew, for instance on ISS when transiting the South Atlantic Anomaly [148]. The atmosphere and magnetosphere of Earth act as a protective layer from space radiation, being beneficial for current LEO missions, but not helpful in the case of interplanetary travel [145]. Even with previous recommendations made by the US National Council on Radiation Protection and Measurements [149,150] and after several decades of research, limited progress has been made to fully understand and diminish the effect of space radiation on astronauts beyond LEO [146].

As the distance from Earth and the duration of missions will increase during interplanetary travel, so does the radiation exposure in the intravehicular environment. This statement also applies to onboard medical kits that may experience drug instability during long-distance spaceflight [147]. The effect of direct ionization on new drug delivery systems has been studied, the sterilization processes leading to radiolysis products [151] and to degradation in polyester carriers in time [152]. Even though it has been evidenced that indirect ionization induces reduced damage in drugs compared to direct ionization, liquid formulations are nonetheless prone to alterations due to less concentrated target molecules than in solid forms. In this case, drug degradation implies free radicals formation, such as oxygen, which results from the breakdown of water in aqueous formulations [147].

Still, little is known about the effect of low doses on drugs in all its complexity. Currently, radiosterilization of pharmaceuticals implies doses in the range of 25 kGy–50 kGy, exceeding by far the ~0.5 Gy that would be accumulated during Martian missions. Providing radiosterilization doses for such short time durations as minutes or hours substantially surpasses the estimated radiation dose-rates amassed over several years of interplanetary travels. It has been put forward that drug stability should be preserved when exposed to low radiation levels, like those anticipated for distant space missions, since at high doses/dose-rates, such as those delivered during radiosterilization, they remain stable. However, many drugs exhibited better stability at increased dose-rates. This observation may be associated with the activity of radical species [147]. The stability of nalidixic acid, spectinomycin and rifampicin exposed to gamma radiation up to 205 kGy has been studied with reference to their UV-Vis and FTIR spectra in Reference [153]. The overall observation was that all investigated antibiotics proved to be sensitive to doses up to 24 kGy, spectinomycin being the most vulnerable to gamma rays. The higher concentration for at least one byproduct that was generated upon radiation exposure is in direct connection with the increase in radiation dose.

Regarding the type of pharmaceutical formulations, solutions are usually acknowledged to be less stable compared to solid or semisolid forms. In particular, water-based drug solutions can lead to frequent and fast degradation reactions, taking into consideration the interaction of drug molecules with reactive species generated by water. Therefore, in Reference [154], only liquid pharmaceutical formulations have been studied in the context of space radiation. It has been supposed that if the vulnerable liquid formulations are found to be stable, then the solid formulations should pose no further concern. In apparent disagreement, there have been several reports, outlined by Blue et al., indicating that even drugs in solid or powder state can exhibit radiation-induced instability [147].

To preserve astronauts’ health and performance during long-duration spaceflights, providing safe, effective and stable pharmaceuticals must be a requisite part of the missions. At the beginning of human space exploration, only three medicines were taken onto the mission [145], but during the early stages of Space Shuttles, more than 500 individual doses of 31 different drugs have been administered, with 83 % of astronauts using at least one medicine [22]. Medical kits on ISS comprise a large number of pharmaceuticals in different formulations alongside diagnostic and therapeutic equipment [145]. Additional reports on medication usage during spaceflights are provided in References [6,155,156]. Antimicrobial agents in ISS medical kits, aside from antibiotics, include antifungal, antiviral and antiparasitic drugs, thus making possible the prevention, treatment and control of a broad spectrum of infections via different routes of administration [8].

Changes in pharmacokinetics (e.g., absorption, distribution, metabolism and excretion of drugs during spaceflight as discussed in Reference [20]) and/or pharmacodynamics [6,20] potentially impact efficacy alongside safety, and on top of that, altered stability has to be accounted when it comes to drug exposure to spaceflight environmental factors [21], such as radiation, microgravity, excessive vibrations, CO_2_-rich atmosphere, humidity and temperature fluctuations [157]. Pharmaceuticals can become unstable through alteration of either their physical (changes in appearance or consistency) or chemical properties (loss of potency, alteration of excipients, interactions of excipients with active ingredients, changes in solubility or generation of toxic degradation products) [6,145]. For drug stability assessment in the outer space environment, it is necessary to establish that no considerable modifications of the active pharmaceutical ingredient (API) of drugs appear following exposure and also no substantial degradation byproducts are formed that can be toxic or can lead to alterations of the properties of the initial compound [158].

Many studies investigated radiation-induced physico-chemical modifications in pharmaceuticals at sterilization equivalent or higher doses (up to several hundred kGy). For example, powder formulations of methylxanthine derivatives, such as caffeine, theophylline and theobromine, have been exposed to ionizing radiation via an electron beam in the dose range of 25 kGy–400 kGy, resulting in no color changes and relatively high radiochemical stability up to sterilization doses [159]. On the contrary, metoclopramide aqueous formulations subjected to both gamma rays and high-energy electrons displayed color changes and generated degradation byproducts [160]. The solid-state β-blockers irradiated by high e-beam also showed color and melting point modifications [161]. As an interesting fact, two cephalosporin antibiotics with similar molecular structures—which would suggest that they might have similar responses to the same radiation sources and doses—exhibit somehow different radiosensitivity, cephradine being highly unstable following gamma radiation, while cefotaxime presents < 0.1 % degradation and high stability [162].

Although studies were also performed on pharmaceuticals that were exposed to the space environment, only a limited number tackled their stability, as shown in Table 1.

In such cases, besides radiation, the impact of different gravitational conditions—micro- and hypergravity—also intervenes. The microgravity exposure—apart from being responsible for alterations in astronauts and microbes—may induce changes in drugs. Du et al. tested physical and chemical changes in time for 35 medicines, representing 18% of the entire kit usually taken onto space voyages, which have been continuously exposed to the space environment on the ISS during 28 months and compared to identical ground controls after the return. The most often encountered physical changes consisted of discoloration. Phase separation within creams and ointments has also been identified. Regarding chemical modifications, an increased number of drugs stored on ISS exhibited lower API content in comparison with terrestrial controls, the number of medicines failing United States Pharmacopeia (USP) API requirements raising as time spent in space expanded. In many cases, the reduction in potency took place before the labeled expiration date. It has been found that cefadroxil, atorvastatin and PMZ in liquid form suffered degradation more than two times quicker when flown on the ISS in contrast to the ones stowed on Earth [157]. Another investigation by Wotring tested the hypothesis that the aging of drugs stored on ISS does not induce uncommon degradation and has found that eight out of nine medicines passed the 2012 USP requirements for API content, with no unusual degradation products being identified [163]. The effect of spaceflight storage on vitamin stability was also investigated by Chuong et al. [164] and Zwart et al. [165].

Even though medicines are designed to be implemented during space missions in microgravity conditions, they would certainly transit hypergravity environments when launched or during landing on another planet. Therefore, it is imperative to know if pharmaceuticals subjected to a high-g environment remain stable prior to administering them to astronauts during flight. In this respect, Simon et al. carried out stability studies on some phenothiazines pre- and post-hypergravity exposure, these photoactive non-antibiotic drugs being chosen due to their multifunctionality when it comes to transformation into antimicrobial agents by laser radiation. Unirradiated and laser-irradiated solutions have been subjected to a gravitational acceleration that surpasses 20 times that experienced at the Earth’s surface by using the European Space Agency’s Large Diameter Centrifuge [166]. Results showed no significant differences in pH between uncentrifuged and centrifuged samples, whereas in the absorption peaks intensity a slight variation was obtained via UV-Vis spectroscopy (without the appearance of new peaks, bands/shoulders or shifts towards shorter/longer wavelengths). Untreated and laser-treated samples exhibited no changes in the characteristic vibrations of their molecular bonds identified within FTIR spectra after being subjected to centrifugation. No differences were evidenced in the number of generated photoproducts and their intensity via thin layer chromatography in the function of gravity levels. Thus, one may state that unirradiated and laser-irradiated phenothiazine solutions remain stable even after being exposed to an increased gravitational environment [167].

Some of the terrestrial approaches proposed to handle MDR-associated challenges, presented in Section 3, could be undertaken during prolonged spaceflights, since commercially available antimicrobial agents may not be stable enough for such ventures.

## 6. Conclusions

This review presents the evolution of different medicines in terrestrial, microgravity and hypergravity conditions in direct connection with their implementation during space missions.

It is shown that deep space exploration may increase the susceptibility and severity of infections. To effectively treat infections encountered in outer space, multidisciplinary studies, concerning immunology, microbiology and pharmacology, are required.

Two major challenges have to be accounted for: space radiation and microgravity whose effect on humans, microorganisms and pharmaceuticals are influenced by the duration and final target of the missions along with the phase of the flight.

A crucial element of space missions hovers around the stability of drugs included in space medical kits. On one hand, radiation-induced instability has been evidenced in many reports devoted to studying such an issue on Earth and in outer space as well. However, of paramount importance is the impact of gravity, since it is questionable if the potency of antimicrobial agents is diminished not only due to the microgravity-induced weakened immune response of astronauts, but also due to the gravity-altered pathogens. Physico-chemical alterations in space-flown drugs have been detected; however, sometimes it is difficult to determine whether the radiation or gravitational environment influenced them.

In the context of such reports, the paper presents also some detailed schemes and procedures to find reliable ways for fighting MDR acquired by microorganisms. It shows that the role of multifunctional medicines modified at the molecular scale by optical methods should be considered in more detail for long-term space missions.

Several papers proposed possible solutions to maintain drug stability even in extreme environmental conditions, such as those that would be encountered during long-duration space exploratory missions, amongst the suggested approaches being the implementation of novel pharmaceutical formulation strategies along with radioprotective packaging and storage.

## Figures and Tables

**Table 1:** Stability studies carried out on drugs subjected to the spaceflight environment. The majority of pharmaceutical agents were in solid formulations, a few semi-solid and liquid formulations being tested as well by Du et al. Space-flown pharmaceuticals have been compared to ground controls (excepting Wotring’s). A subset of terrestrial samples from Chuong et al. have also been exposed to heavy ion and proton radiation.

Drug	Formulation	Spaceflight Duration	Stability Criterion	Study
acetaminophen *^,^** aspirin ibuprofen loperamide *** loratadine *^,^**^,^*** melatonin * modafinil *** pseudoephedrine zolpidem ***	solid	550 days	API content degradation products	Wotring [163]
acyclovir * amoxicillin/clavulanate * atorvastatin azithromycin cefadroxil ciprofloxacin * clotrimazole * cobalamine dextroamphetamine * epinephrine * fluconazole * furosemide * ibuprofen imipenem/cilastatin * levofloxacin * levothyroxine * lidocaine * metoprolol succinate * metronidazole mupirocin * phenytoin * progestin/estrogen * promethazine * risedronate * sertraline * silver sulfadiazine * sulfamethoxazole/trimethoprim * temazepam * triamcinolone *	solid semi-solid liquid	14 days 353 days 596 days 880 days	API content physical propertieschemical properties	Du et al. [157]
Centrum Silver^®^ multivitamin Once A Day^®^ women’s multivitamin *	solid	14–20 days 12–19 months	API content (B complex only)	Chuong et al. [164]
Centrum Silver^®^ multivitamin **** Nature’s Way^®^ vitamin D supplement ****	solid	13 days 353 days 596 days 880 days	API content	Zwart et al. [165]

* Pharmaceuticals exhibiting alterations in API and physical/chemical properties or having increased degradation products/impurities in one or more formulations. ** Pharmaceuticals passing the acceptance criteria for API content at the time of tests, but failing under updated guidelines. *** Pharmaceuticals containing unidentified/unspecified impurity products. **** Multivitamins exhibiting time-associated instability without spaceflight-correlated modifications.

## Data Availability

Not applicable.
